# The rate of and risk factors for falls among patients with cirrhosis: A multicenter study

**DOI:** 10.1097/HC9.0000000000001019

**Published:** 2026-08-20

**Authors:** Joy E. Obayemi, Mitchell Paukner, Bima J. Hasjim, Dominic Vitello, Paola A. Barrios, Saad Hussain, Salva Balbale, Lisa B. VanWagner, Mohsen Mohammadi, Sanjay Mehrotra, Oriana Dentici, Lihui Zhao, Daniela P. Ladner

**Affiliations:** 1 Northwestern University Transplant Outcomes Research Collaborative (NUTORC), Comprehensive Transplant Center (CTC), Northwestern University Feinberg School of Medicine, Chicago, Illinois, USA; 2 Department of Biostatistics and Data Science, Wake Forest University, Winston-Salem, North Carolina, USA; 3 Department of Medicine, Division of Gastroenterology and Hepatology, Northwestern University Feinberg School of Medicine, Chicago, Illinois, USA; 4 Department of Surgery, Northwestern University Feinberg School of Medicine, Center for Health Services and Outcomes Research, Institute of Public Health and Medicine & Northwestern Quality Improvement, Research, & Education in Surgery (NQUIRES), Chicago, Illinois, USA; 5 Center of Innovation for Complex Chronic Healthcare (CINCCH), Edward Hines, Jr. VA Hospital, Hines, Illinois, USA; 6 Department of Medicine, Division of Digestive and Liver Diseases, University of Texas Southwestern Medical Center, Dallas, Texas, USA; 7 Department of Industrial Engineering and Management Sciences, Northwestern University, Evanston, Illinois, USA; 8 Department of Preventive Medicine, Northwestern University, Chicago, Illinois, USA; 9 Department of Surgery, Division of Transplantation, Northwestern Medicine, Chicago, Illinois, USA

**Keywords:** frailty, frequency, health services outcomes research, liver disease, morbidity

## Abstract

**Background:**

Falls are associated with frailty and are a significant cause of morbidity among the elderly. Due to accelerated physiologic aging, frailty is common among patients with cirrhosis. We sought to identify the patients with cirrhosis at highest risk of falling and to explore the relationship between falls and frailty in this population.

**Methods:**

We conducted a longitudinal, retrospective analysis of adults (above 18 y old) with cirrhosis from 2011 to 2021 in the multi-institutional CAPriCORN database. Falls were defined by validated International Classification of Diseases 9th and 10th edition codes. Frailty was defined using the Hospital Risk Frailty Score (mild <5, intermediate 5–15, severe >5). Multivariable Cox models identified risk factors for experiencing a first and second fall (HR: 95% CI).

**Results:**

In total, 42,046 patients with cirrhosis were included with a median (IQR) age of 59.3 years (51.4–66.9) and mean (IQR) follow-up of 3.4 years (0.6–5.7). In total, 58.0% were male (n=24,390) and 59.5% had baseline compensated cirrhosis (n=25,021). Overall, 7.1% (n=2997) experienced a fall within the study period, 2.6% within 1 year of cirrhosis diagnosis. Prior fall history (HR: 5.69, 4.93–6.58), severe frailty (HR: 2.37, 2.02–2.80), and intermediate frailty (HR: 1.71, 1.49–1.96) were the strongest fall risk factors. In total, 38.4% of patients who fell experienced an additional fall during follow-up, 35% of which occurred within 1 year. Fall history (HR: 1.50, 1.18–1.90) and severe frailty (HR: 1.51, 1.16–1.96) were the strongest risk factors for a second fall. Patients with severe frailty had a 10-year cumulative fall incidence of 19.6%.

**Conclusions:**

Though 7% of patients experienced a fall, patients with a fall history or frailty were at significantly increased risk. These findings may help target patients with the highest fall risk and design fall-prevention interventions for this uniquely vulnerable population.

## Graphical abstract

**Figure:**
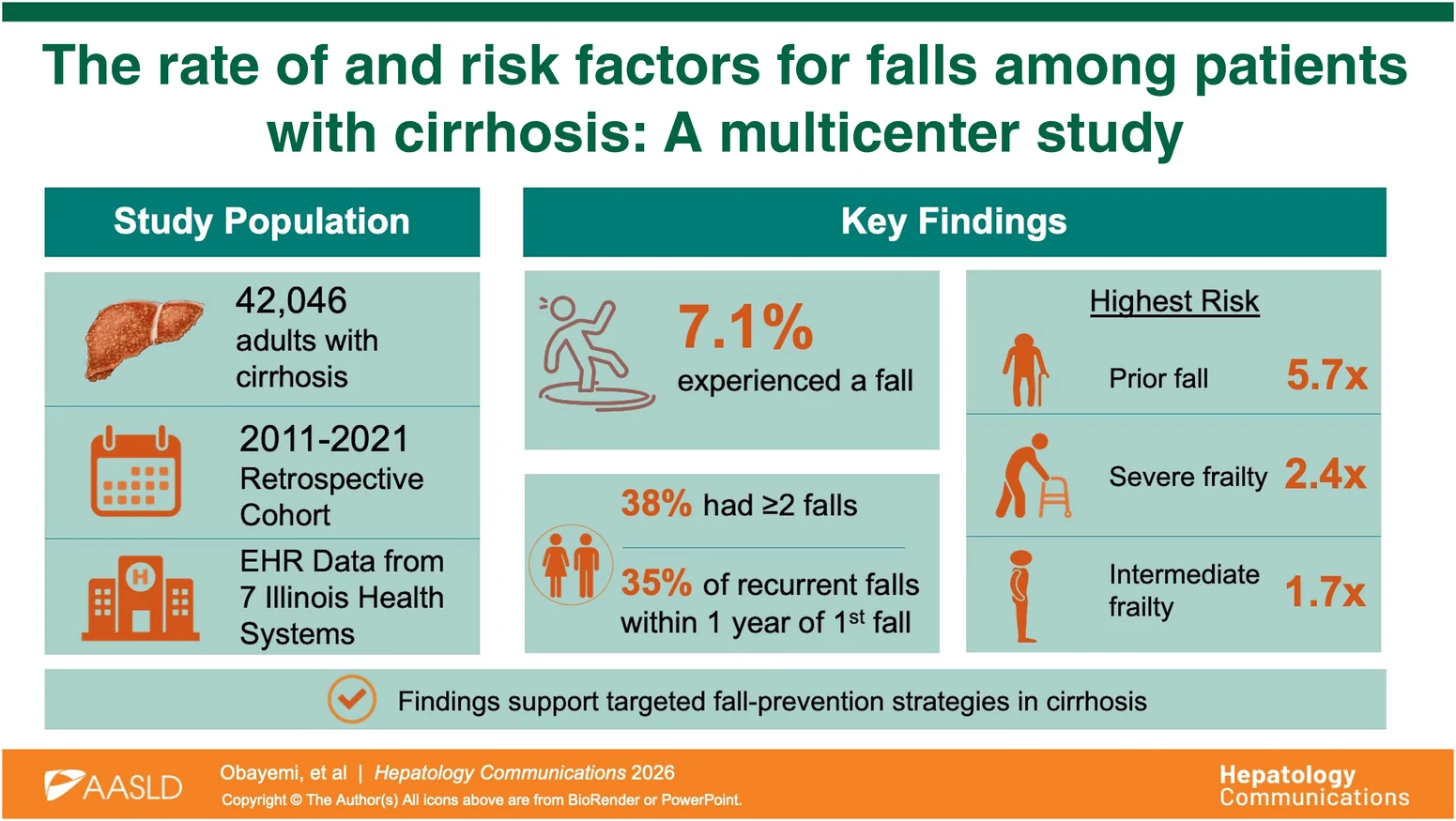

## INTRODUCTION

Each year, 1 in 3 adults over 65 years old experiences at least one fall,^1^ generating approximately 3 million emergency room visits annually.^2^ Falls have been independently associated with subsequent functional decline in the elderly.^3^ The number of falls experienced by older adults is increasing, and mortality associated with falls in patients 65 years old or above increased by 41% from 2012 to 2021.^4^ As the American population continues to age and the frequency of falls increases, morbidity, mortality, and costs of falls represent a mounting public health concern.

While age is a key risk factor for falls, increasingly prevalent chronic diseases such as cirrhosis can accelerate physiologic aging and increase risks of falls and related injuries^5^,^6^,^7^,^8^ Patients with cirrhosis experience increased physiologic frailty due to osteoporosis, hypogonadism, protein malnutrition, and other physiologic derangements associated with liver dysfunction.^9^ Kremer and colleagues found that a high Clinical Frailty Score (>4) was independently associated with increased mortality (HR: 1.53), and Hasjim and colleagues found that hospitalization rates were 5 times higher among compensated patients with cirrhosis and severe frailty (OR: 5.2).^10^,^11^ However, the association between frailty and fall risk in this population has only been investigated in a single prospective study (n=299).^12^

Prior work suggests that investigating the clinical impact of falls on patients with cirrhosis may allow for targeted interventions to reduce frailty-related morbidity by preventing falls.^9^ However, few have specifically examined risk factors for falls in this population in the current clinical landscape. As cirrhosis prevalence increases and the population ages, studies that leverage the power of large datasets can identify key risk factors for falls and fall recurrence. We used a large, longitudinal dataset to retrospectively assess (1) the annual and cumulative incidence of falls in patients with cirrhosis, (2) key clinicodemographic factors associated with high risk for falls, and (3) the association between fall risk and frailty. We hypothesized that intermediate and severe frailty would be associated with an increased risk of experiencing a first and second fall.

## METHODS

### Study design and data source

We conducted a retrospective cohort study of patients in the Chicago Area Patient-Centered Outcomes Research Network (CAPriCORN) database from January 1, 2011, to August 1, 2021. CAPriCORN is a clinical data repository of electronic medical records (EMR) from over 12.8 million patients at 32 hospitals in the Chicagoland area.^13^,^14^ CAPriCORN data includes demographic and clinical data (International Classification of Diseases 9th and 10th editions (ICD-9/ICD-10), Current Procedural Terminology (CPT) codes, medications, laboratory results). This study was conducted in accordance with both the Declaration of Helsinki and Istanbul. The studyfollowed the Strengthening the Reporting of Observational Studies in Epidemiology (STROBE) guidelines and was approved by the Northwestern University Institutional Review Board (IRB ID # STU00213447). Given the use of de-identified aggregate data, the need for informed consent was waived.

### Study cohort

Adult patients (above 18 y old) with a cirrhosis diagnosis were included in the study cohort. Diagnosis of cirrhosis was based on ICD-9 and ICD-10 codes using previously published algorithms^10^,^15^ (Supplemental Table S1, https://links.lww.com/HC9/C454). Patients were excluded from analyses if they were below 18 years old (n=3; <0.01%), had a missing inclusion date (n=217; 0.5%), or no follow-up data after the first encounter (n=2842; 6.8%).

### Clinicodemographic covariates

The first appearance of a cirrhosis code or cirrhosis-related decompensation event was the cirrhosis index date. Patient demographics at cirrhosis index date included age, sex, self-reported race/ethnicity, type of health insurance, etiology of disease, decompensation status, MELD-Na, frailty, fall history prior to cirrhosis diagnosis, and HCC status. Race/ethnicity was categorized based on established guidance from the US Office of Management and Budget’s Revisions to the Standards for the Classification of Federal Data on Race and Ethnicity to include Asian, Hispanic, non-Hispanic Black, and non-Hispanic White.^16^ Additional races, including American Indian or Alaska Native, Pacific Islander, multiracial, and a patient-reported “other” race, were aggregated into the “Other” race category to enable meaningful analysis. Insurance status was categorized into Medicare/Medicaid (which includes those with “government” insurance), private insurance, no insurance (ie, self-pay), and other insurance status (including unknown).

Cirrhosis etiology was identified from specific ICD-9/-10 codes using validated algorithms from published work.^6^,^10^,^17^ In accordance with recent AASLD (American Association for the Study of Liver Disease) guidelines for liver disease nomenclature, patients were divided into the following mutually exclusive categories: alcohol-associated liver disease (ALD), metabolic dysfunction–associated steatotic liver disease (MASLD), metabolic dysfunction with increased alcohol intake (MetALD), HBV, HCV, ≥2 etiologies, and other (eg, biliary cirrhosis, genetic liver disease, cardiac etiology).^18^ MASLD was defined by having ICD-10 codes for NAFLD/NASH cirrhosis or—if another cirrhosis etiology or NAFLD/NASH diagnosis was absent—by having (1) an ICD-9/-10 code for a liver disease–associated cardiometabolic risk factor or (2) a prescription for a medication that treats any cardiometabolic risk factors.^18^,^19^ MetALD was defined by having ALD plus criteria for a MASLD diagnosis.^19^

Decompensated cirrhosis was defined as the occurrence of ascites, spontaneous bacterial peritonitis, HE, variceal bleeding, hepatorenal syndrome, or hepatopulmonary syndrome using validated ICD-based, CPT-based, and medication-based algorithms (Supplemental Table S1, https://links.lww.com/HC9/C454).^20^,^21^,^22^ Patients were considered to have compensated cirrhosis at baseline if they did not have any decompensation event within 3 months of their cirrhosis index date. Patients with HE at baseline were included in the decompensation group as well as independently described because of the known association between encephalopathy and the propensity to fall.^23^ MELD-Na Score was calculated within 90 days of the cirrhosis index date; the highest score was used if multiple scores were available during this time frame.^24^

Frailty was classified at cirrhosis index date using the Hospital Risk Frailty Score (mild<5, intermediate 5–15, severe >15)—a validated claims-based score derived from 109 ICD-10 codes that capture challenges with mobility, functional dependence, cognitive impairment, anxiety and depression, incontinence, and other chronic conditions.^25^,^26^ The HFRS has long been considered a multidimensional measure of global frailty that corresponds closely with objective measures of frailty, such as the Fried Frailty Index and Rockwood Frailty Index.^25^,^27^ Although the HFRS was created in an elderly inpatient cohort, it has since been applied to a number of settings and patient populations, including cirrhosis.^28^ Additional frailty markers that are more specific to the cirrhosis population were incorporated, including osteoporosis, sarcopenia, and malnutrition. A patient was considered to have a fall history if a validated code for a fall was present prior to the cirrhosis index date. HCC status was determined using validated ICD-9 and ICD-10 codes.^29^ The Charlson Comorbidity Index (CCI) was used to assess the global burden of comorbid conditions, and patients were categorized by score into having no (0), mild (1–2), moderate (3–4), or severe comorbidities (≥5) based on documented clinical diagnoses on the cirrhosis index date. Individual clinical characteristics were identified and described, including coronary artery disease, cerebrovascular disease, congestive heart failure, moderate or severe kidney disease, diabetes mellitus, non-HCC malignancy, psychological disorders (including cognitive and substance use disorders), and dementia (Supplemental Table S2, https://links.lww.com/HC9/C454]. Baseline use of high-risk medication classes, such as diuretics, opioids, benzodiazepines, and antidepressants, was identified [Supplemental Table S2, https://links.lww.com/HC9/C454]. Baseline use was defined as having at least 1 refill for a prescribed medication within the class in the 90 days following the cirrhosis index date.

### Study outcomes

The primary outcome was a clinically documented fall after the cirrhosis index date. Falls were identified using validated ICD-9/-10 codes (Supplemental Table S3, https://links.lww.com/HC9/C454].^1^,^2^,^7^ Patients with falls prior to or on the cirrhosis index date were categorized as having a history of falls, and those were not counted as falls after cirrhosis diagnosis. Multiple fall codes on the same date were the same fall event. Falls over time were reported as falls per patient per year (pppy). Falls were categorized as injurious if they were associated with an injury ICD-9 or ICD-10 code within 30 days of the documented fall date (Supplemental Table S3, https://links.lww.com/HC9/C454]. Prior published work describes identifying fall-related injuries anywhere from 3 weeks to 2 years after a fall.^30^,^31^ Given the known variability in accuracy of administrative coding, this 60-day window was chosen as a time frame where the most significant injuries would be documented. Similarly, a hospitalization-associated fall was defined as a fall documented within 30 days of a hospitalization. Lastly, inpatient falls were defined as falls that took place at least 1 day following a hospital admission up until the day of discharge.

### Statistical analysis

We calculated the cumulative incidence of falls, deaths, and transplants in patients with cirrhosis, creating cumulative incidence curves stratified by frailty, cirrhosis etiology, baseline compensation status, age, CCI category, and MELD-Na score to depict the burden of falls over time via univariable analysis. Our primary analysis was performed on the entire cohort and evaluated the relationship between different clinicodemographic covariates among patients who did or did not experience a fall (no falls vs. ≥1 fall during the study period). Descriptive statistics (ie, counts and percentages; mean, median, and IQR) were used to describe the demographic and disease characteristics of patients based on fall status. For this analysis, the start of observation (time=0) was set as the index date of cirrhosis diagnosis. To determine if the relationship between covariates and falls was significant, an omnibus χ^2^ test was used for categorical variables, and *t* tests were used to compare continuous variables. If the omnibus χ^2^ test produced a *p* value below the level of significance (alpha=0.05), a collection of pairwise tests with Bonferroni adjustments was performed to identify which levels of the categorical variable were different across second fall status. Fall characteristics (ie, injurious falls and associated hospitalizations) were reported using counts and cohort percentages.

Secondary analysis was performed utilizing the cohort of patients who experienced ≥1 fall during the study period to evaluate the effect of different covariates on the occurrence of a second fall, where the start of observation was set as the date of the first documented fall. The relationship between covariates and second fall status was evaluated in the same fashion as the primary analysis.

In both the primary and secondary cohorts, multivariable cause-specific Cox proportional hazards regression analyses—or cause-specific hazards models—were performed to estimate the fall-specific HR associated with baseline covariates, accounting for the competing risks of death and transplant events. A sensitivity analysis was conducted using a Fine-Gray model for competing risks, which showed nearly identical results (Supplemental Table S6, https://links.lww.com/HC9/C454, Supplemental Table S7, https://links.lww.com/HC9/C454]. These models were adjusted for age at cirrhosis index date, sex, self-reported race and ethnicity, type of health insurance, etiology of disease, baseline decompensation status, frailty, fall history prior to cirrhosis diagnosis, HCC status, CCI, malnutrition, sarcopenia, osteoporosis, and baseline use of high-risk medications. The estimated HRs were reported with 95% CIs. Two-sided *p* values were calculated for all results, and the threshold for statistical significance was 0.05 based on the *p* values. Data analysis was performed using R Studio (version 4.3.1).

## RESULTS

### Overview

A total of 42,046 patients with cirrhosis were included in the study cohort, with a median (IQR) age of 59.3 years (51.4–66.9) and a median (IQR) follow-up time of 2.5 years (0.62–5.74). In the cohort, 42.0% (n=17,656) were female, 44.9% (n=18,897) were non-Hispanic White, 21.6% (9097) were non-Hispanic Black, and 19.5% (n=8199) were Hispanic. Patients were insured predominantly through Medicare/Medicaid (n=19,850, 47.2%) and private plans (n=10,767, 25.6%), while 7.7% (n=3229) of the cohort did not have insurance and 19.5% (n=8200) had an “other” or unknown form of insurance. Etiology of cirrhosis was most commonly MASLD (n=9863, 23.5%), MetALD (n=8321, 19.8%), and patients with ≥2 etiologies (n=7183, 17.1%). At baseline, 59.5% (n=25,021) of patients were compensated. In total, 14.0% (n=5872) of patients had documented HE. MELD-Na scores were available for 60.5% of patients in the cohort, with a median of 16 (IQR: 10–24). In total, 64.2% (n=26,978) of patients had mild frailty, 23.7% (n=9945) had intermediate frailty, and 12.2% (n=5123) had severe frailty at baseline. In all, 5.5% (n=2323) had a history of HCC (Table 1). Rates of additional comorbidities are described in Table 2. Median CCI score for the overall cohort was 8 (IQR: 4–11), which is categorized as having severe comorbidities. In total, 5.8% (n=2456) patients were malnourished, 2.6% (n=1112) had osteoporosis, and 0.15% (n=61) had sarcopenia. Regarding baseline medication use, 17.7% (n=7450) chronically used diuretics, 2.8% (n=1196) used opioids, 17.2% (n=7214) used benzodiazepines, and 23.4% (n=9833) used antidepressants (Table 2).

**TABLE 1:** Patient demographics comparing patients with ≥1 fall and those with no documented falls

Demographics	Total cohort	Patients with ≥1 fall	% (by row)	Patients with no falls	% (by row)	*p*
Total (n)	42,046	2997	7.1	39,049	92.9	
Age, y (mean) {median} [IQR]	(58.8){59.3} [51.4–66.9]	(60.8){60.9} [52.9–69.1]		(58.6){59.2} [51.3–66.8]		<0.001
Age (categorical)						<0.001
[18–55)	14,570	913	6.3	13,657	93.7	
[55–65)	14,387	972	6.8	13,415	93.2	
[65–75)	8828	706	8.0	8122	92.0	
75+	3954	384	9.7	3570	90.3	
NA	307	22	7.2	285	92.8	
Follow-up time, years (mean) {median} [IQR]	(3.4){2.48} [0.62–5.74]	(4.8){4.53} [1.98–7.20]		(3.3){2.34} [0.56–5.58]		<0.001
Sex (N)						<0.001
Men	24,390	1615	6.6	22,775	93.4	
Women	17,656	1382	7.8	16,274	92.2	
Race						<0.001
Asian	1241	64	5.2	1177	94.8	
Hispanic	8199	712	8.7	7487	91.3	
Non-Hispanic Black (NHB)​	9097	677	7.4	8420	92.6	
Non-Hispanic White (NHW)	18,897	1425	7.5	17,472	92.5	
Other	4612	119	2.6	4493	97.4	
Health insurance status						<0.001
Private	10,767	642	6.0	10,125	94.0	
Medicare/Medicaid	19,850	1668	8.4	16,724	91.6	
No insurance	3229	247	7.6	2982	92.4	
Other	8200	440	5.4	7760	94.6	
Etiology						<0.001
MASLD	9863	618	6.3	9245	93.7	
MetALD	8321	864	10.4	7457	89.6	
ALD	3956	170	4.3	3786	95.7	
HCV	6358	321	5.1	6037	95.0	
HBV	996	54	5.4	942	94.6	
Two or more	7183	746	10.4	6437	89.6	
Unspecified	1457	7	0.5	1450	99.5	
Other	3912	217	5.6	3695	94.5	
Compensation status						<0.001
Compensated at baseline	25,021	1603	6.41	23,418	93.59	
Decompensated at baseline	17,025	1394	8.19	15,631	91.81	
HE status						<0.001
HE at baseline	5872	487	8.29	5385	91.71	
No HE at baseline	36,174	2510	6.94	33,664	93.06	
MELD-NA (median) {IQR} [% with score]	(16) {10–24} [60.5]	(16) {10–24} [63.8]		(16) {10–24} [60.2]		
Frailty						<0.001
Mild	26,981	1406	5.2	25,575	94.79	
Intermediate	9946	906	9.1	9040	90.89	
Severe	5123	685	13.4	4438	86.63	
Fall history	1191	400	33.6	791	66.41	<0.001
HCC	2323	69	3.0	2254	97.0	<0.001

Abbreviations: ALD, alcohol-associated liver disease; MASLD, metabolic dysfunction–associated steatotic liver disease; MetALD, metabolic dysfunction with increased alcohol intake.

**TABLE 2:** Comorbidities of the patient cohort comparing patients who fell to others

Demographics	Total cohort (N=42,046)	Patients with ≥1 fall (N=2997)	Patients with no falls (N=39,049)	*p*
Charlson Comorbidity Index (CCI)				
Continuous, median (IQR)	8 (4–11)	9 (6–13)	7 (4–11)	<0.001
0: no comorbidities, N (col %)	283 (0.67)	20 (0.67)	263 (0.67)	<0.001
1–2: mild, N (col %) comorbidity	6401 (15.2)	284 (9.5)	6117 (15.7)	
3–4: moderate comorbidity (col %)	5304 (12.6)	290 (9.7)	5014 (12.8)	
≥5: severe comorbidity (col %)	30,058 (71.5)	2403 (80.2)	27,655 (70.8)	
Specific comorbidities, N (col %)				
Coronary artery disease	5531 (13.2)	593 (19.8)	4938 (12.6)	<0.001
Cerebrovascular disease	1731 (4.1)	210	1521	<0.001
Congestive heart failure	4473 (10.6)	492 (16.4)	3981 (10.2)	<0.001
Moderate or severe kidney disease	2718 (6.5)	240 (8.0)	2478 (6.3)	<0.001
Diabetes mellitus	10,611 (25.2)	1015 (33.9)	9596 (24.6)	<0.001
Non-HCC cancer	5093 (12.1)	410 (13.7)	4683 (12.0)	0.007
Psychological disorder/cognitive disorder/substance use disorder	15,916 (37.9)	1556 (51.9)	14,360 (36.8)	<0.001
Dementia	897 (2.1)	111 (3.7)	786 (2.0)	<0.001
Osteoporosis	1112 (2.6)	163 (5.4)	949 (2.4)	<0.001
Malnutrition	2456 (5.8)	278 (9.3)	2178 (5.6)	<0.001
Sarcopenia	61 (0.15)	5 (0.17)	56 (0.14)	0.9
Medications, N (col %)				
Baseline diuretic use	7450 (17.7)	776 (25.9)	6674 (17.1)	<0.001
Baseline opioid use	1196 (2.8)	116 (3.9)	1080 (2.8)	0.13
Baseline benzodiazepine use	7214 (17.2)	878 (29.3)	6336 (16.2)	<0.001
Baseline antidepressant use	9833 (23.4)	995 (33.2)	8838 (22.6)	<0.001

### Falls

In the study cohort, 7.1% (n=2997) of patients experienced at least one fall during a median of 2.5 years of follow-up; 2.6% of patients experienced a fall within 1 year of cirrhosis diagnosis. The median (IQR) age of patients with at least one fall was 60.9 (52.9–69.1) years old compared to 59.2 (51.3–66.8) years old among patients without a fall (*p*<0.001) (Table 1). Falls were more common among women than men (7.8% vs. 6.6%, *p*<0.001) and among patients with Medicaid/Medicare insurance than the privately insured (8.4% vs. 6.0%, *p*<0.001). A greater proportion of patients with MetALD (10.4%) and ≥2 cirrhosis etiologies (10.4%) experienced a fall compared to patients with MASLD (6.27%), HCV (5.05%), ALD (4.30%), and HBV (5.42%) (*p*<0.001 for pairwise comparisons). Falls were more common among patients with decompensated cirrhosis at baseline than those with compensated cirrhosis (8.19% vs. 6.47%, *p*<0.001), and patients with HE at baseline were more likely to experience a fall than those without HE (8.29% vs. 6.94%, *p*<0.001). In the entire cohort, 2.8% (n=1191) of patients had a history of falls prior to cirrhosis diagnosis. Of these patients, 33.6% (n=400) of patients fell after cirrhosis diagnosis. Patients with severe comorbidities (CCI ≥ 5) were more likely to experience a fall (7.99%) compared to those with mild comorbidities (4.44%) or moderate comorbidities (5.47%) (*p*<0.001 for pairwise comparisons).

Patients who fell 1 or more times were more likely to have documented malnutrition (9.27% vs. 5.58%, *p*<0.001), more likely to have a history of coronary artery disease (19.79% vs. 12.65%, *p*<0.001), cerebrovascular disease (7.01% vs. 3.90%, *p*<0.001), congestive heart failure (16.42% vs. 10.19%, *p*<0.001), moderate or severe kidney disease (8.01% vs. 6.35%, *p*<0.001), diabetes mellitus (33.87% vs. 24.57%, *p*<0.001), non-HCC cancer (13.68% vs. 11.99%, *p*=0.007), psychological disorders (51.92% vs. 36.77%, *p*<0.001), dementia (3.70% vs. 2.01%, *p*<0.001), and osteoporosis (5.44% vs. 2.43%, *p*<0.001) (Table 2). Patients who fell were more likely to have baseline use of diuretics (25.89% vs. 17.09%, *p*<0.001), benzodiazepines (29.30% vs. 16.23%, *p*<0.001), and antidepressants (33.20% vs. 22.63%, *p*<0.001).

### Incidence of falls over time

The rate of falls was 0.33 falls pppy. Among the different cirrhosis etiologies, patients with ALD had the highest fall rate and more than those with HBV, HCV, and other etiologies (ALD 0.80 pppy vs. HCV 0.08 pppy, *p*=0.005; vs. HBV 0.09, *p*=0.007; vs. Other 0.06, *p*=0.004) (Table 3). Patients with a history of falls prior to cirrhosis diagnosis experienced 8.28 falls pppy compared to 0.1 falls pppy among those with no history of prior falls (*p*=0.04).

**TABLE 3:** Summary of fall rate in falls per patient per year (PPPY) by cohort and patient demographics

Demographics	Total cohort	Falls pppy	*p*	Total cohort with ≥1 fall	Falls pppy	*p*
Total (n)	42,046	0.33		2997	1.63	
Age			0.535			0.378
[18–55)	14,570	0.22		913	2.29	
[55–65)	14,387	0.28		972	1.15	
[65–75)	8828	0.66		706	1.5	
75+	3954	0.23		384	1.27	
NA	307	0.12		22	6.06	
Sex (n)			0.567			0.451
Men	24,390	0.27		1615	1.82	
Women	17,656	0.42		1382	1.41	
Race			0.913			0.235
Asian	1241	0.05		64	0.47	
Hispanic	8199	0.28		712	1.61	
Non-Hispanic Black	9097	0.32		677	2.74	
Non-Hispanic White	18,897	0.43		1425	1.25	
Other	4612	0.12		119	0.71	
Health insurance			0.266			<0.001
Private	10,767	0.09		642	0.64	
Medicare/Medicaid	19,850	0.51		1668	1.41	
No insurance	3229	0.69		247	6.50	
Other	8200	0.07		440	1.26	
Etiology			0.739			0.296
MASLD	9863	0.56		618	0.79	
MetALD	8321	0.24		864	2.01	
ALD	3956	0.80		170	4.04	
HCV	6358	0.08		321	2.4	
HBV	996	0.09		54	0.69	
Two or more	7183	0.34		746	1.31	
Unspecified	1457	0.03		7	0	
Other (genetic, cardiac, cholestatic, other)	3912	0.06		217	1.09	
Compensation status			0.133			0.352
Compensated at baseline	25,021	0.16		1603	2.62	
Decompensated at baseline	17,025	0.58		1394	2.92	
HE status			0.304			0.348
HE at baseline	5872	0.99		487	3.13	
No HE at baseline	36,174	0.22		2510	2.69	
Frailty			0.001			0.529
Mild (<5)	26,978	0.09		1406	1.9	
Intermediate (5–15)	9945	0.47		906	1.6	
Severe (>15)	5123	1.35		685	1.12	
Fall history			0.039			0.746
No	40,855	0.10		2597	1.67	
Yes	1191	8.28		400	1.4	
HCC						
No	39,723	0.35	0.032	2928	1.58	0.388
Yes	2323	0.08		69	4.05	

Abbreviations: ALD, alcohol-associated liver disease; MASLD, metabolic dysfunction–associated steatotic liver disease; MetALD, metabolic dysfunction with increased alcohol intake; pppy, per patient per year.

**FIGURE 1:**
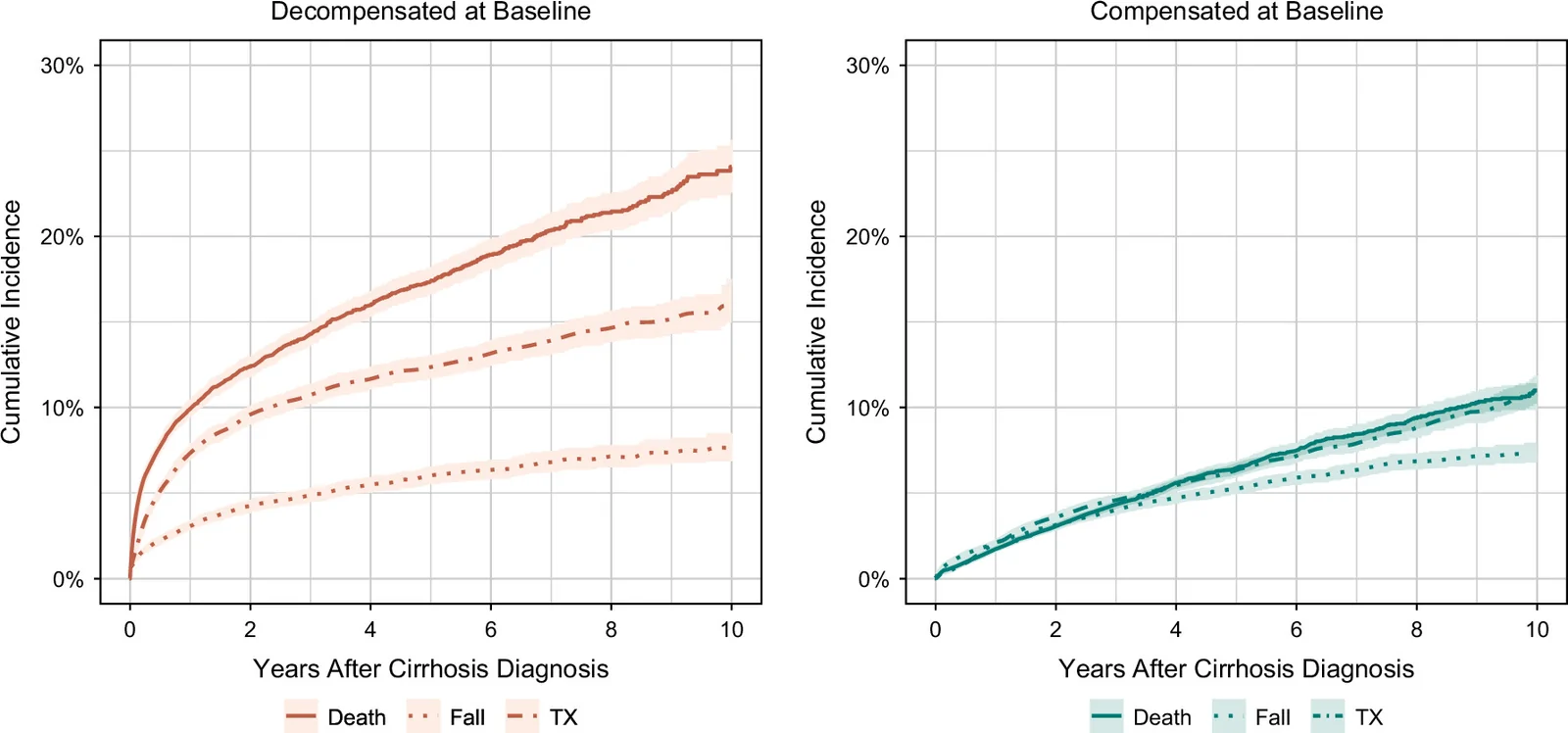
Cumulative incidences of fall, death, and transplant stratified by baseline decompensation status. Abbreviation: TX, transplant.

**FIGURE 2:**
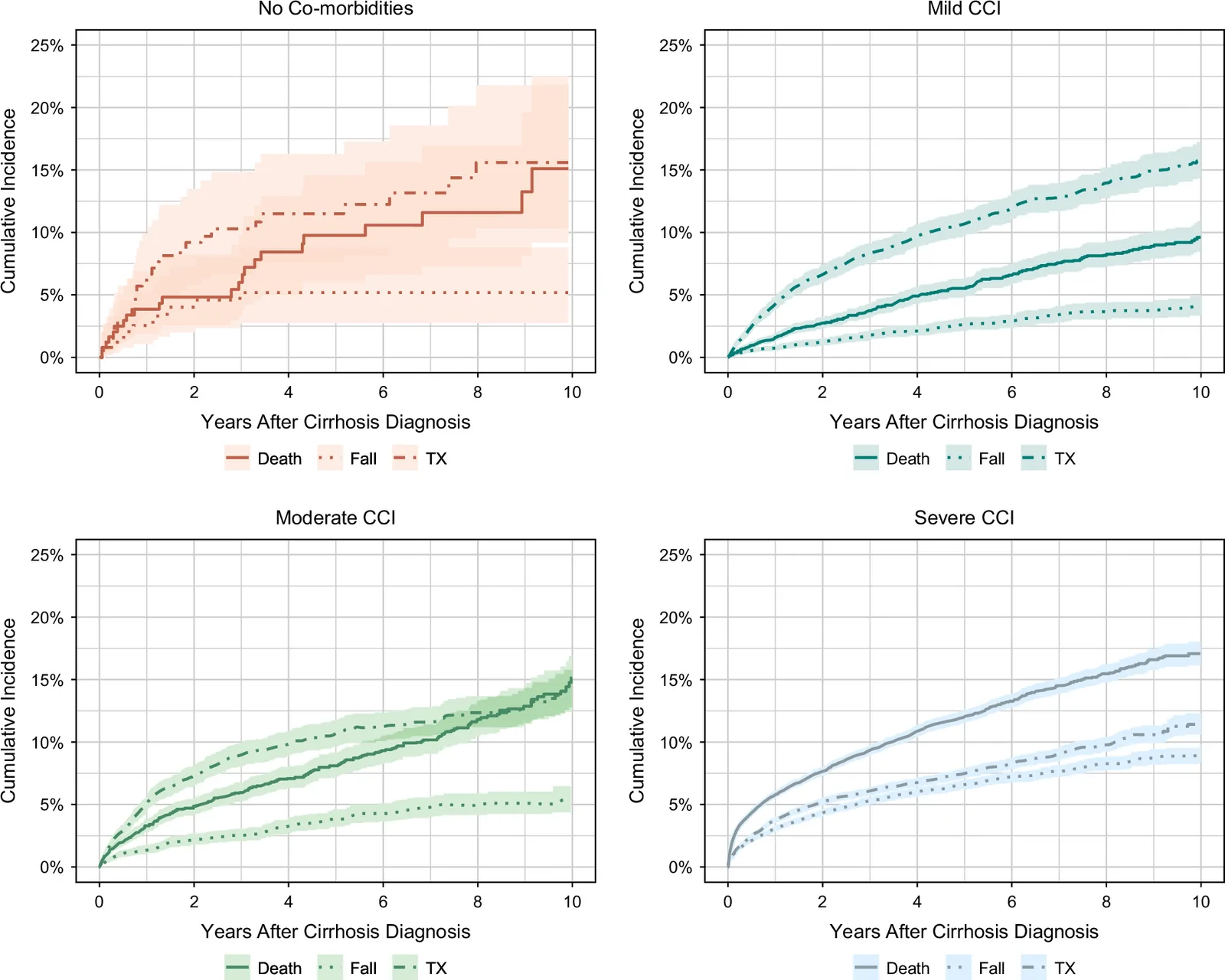
Cumulative incidences of fall, death, and transplant stratified by Charlson Comorbidity Index category. Abbreviation: CCI, Charlson Comorbidity Index; TX, transplant.

Stratifying by decompensation status, the 1-year cumulative incidence of falls among patients with baseline decompensation was 3.1% vs 2.1% for compensated patients (*p*<0.05) (Figure 1). However, in years 3, 5, and 10, there is no significant difference in cumulative fall incidence based on baseline decompensation status (Figure 1). When stratified by CCI category, patients with severe comorbidities had significantly higher cumulative fall incidence at 1 (3.1%), 3 (5.4%), 5 (6.8%), and 10 years (9.2%) of follow-up compared to other CCI categories (all pairwise *p* values <0.001) (Figure 2). In a subanalysis stratified by cirrhosis etiology, cumulative fall incidence was found to be highest for patients with MetALD and ALD [Supplemental Figure S1, https://links.lww.com/HC9/C454]. This pattern was consistent throughout the follow-up period, with a 5-year cumulative fall incidence of 7.5% (95% CI: 6.8%–8.3%) for patients with MetALD and 6.2% (95% CI: 5.0%–7.6%) for patients with ALD. When stratified by MELD, patients with baseline MELD>15 had a significantly higher cumulative fall incidence at 1-year (3.7%, 95% CI: 3.3%–4.1%) and 3-year (5.4%, 95% CI: 4.9%–5.9%) follow-up compared to those with baseline MELD≥15 (*p*<0.0001) (Supplemental Figure S2, https://links.lww.com/HC9/C454]. No significant difference was seen at the 5-year and 10-year mark. When stratified by age category, patients 75 years or older had a higher cumulative fall incidence than other age groups throughout the follow-up period (Supplemental Figure S3, https://links.lww.com/HC9/C454].

### Fall characteristics

In assessing the characteristics of the first fall patients in this cohort, 6.1% (n=187) of falls occurred while patients were inpatients at hospitals, while the rest occurred in outpatient settings. Most falls were not associated with hospitalizations (58.4%, n=1750), but were associated with an injury (80.9%, n=2425) (Table 4). Regarding mortality, 18 patients (0.6%) died within 3 months of experiencing a fall, and 35 patients (1.2%) died within 12 months.

**TABLE 4:** First fall characteristics

First fall characteristics	N (total = 2997)	Percent of first falls
Setting		
Inpatient	187	6.1
Outpatient	2810	93.8
Associated hospitalization		
Yes	1247	41.61
No	1750	58.39
Injury		
Injurious fall	2425	80.91
Noninjurious fall	572	19.09

### Risk factors for falls

**FIGURE 3:**
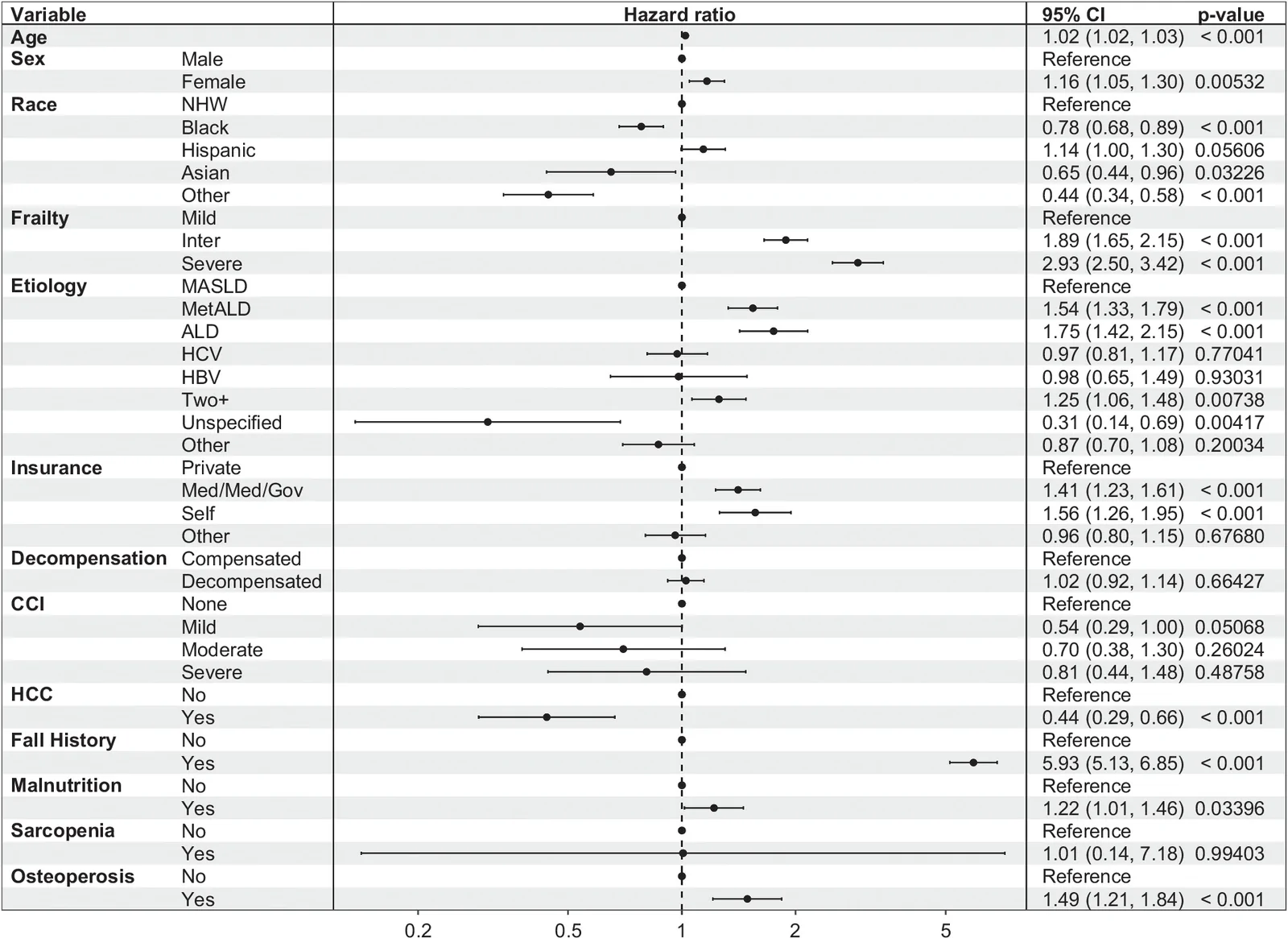
Multivariable cause-specific Cox proportional hazard regression with Forest Plot assessing risk factors for experiencing a first fall. Abbreviations: CCI, Charlson Comorbidity Index; NHW, non-Hispanic White.

After adjusting for clinicodemographic factors, women had a 18% higher risk of experiencing a fall in the study period compared to men (HR: 1.15, 95% CI: 1.03–1.28) (Figure 3). Compared to patients with MASLD, patients with MetALD had a 47% increased risk of experiencing a fall (HR: 1.47, 95% CI: 1.26–1.71, *p*<0.001), patients with ALD had a 68% increased risk of experiencing a fall (HR: 1.68, 95% CI: 1.36–2.07, *p*<0.001), and patients with ≥2 cirrhosis etiologies had a 22% increased risk of experiencing a fall in the study period (HR: 1.22, 95% CI: 1.04–1.44, *p*=0.017). Patients with Medicare/Medicaid insurance had a 40% higher fall risk than patients with private insurance (HR: 1.40, 95% CI: 1.23–1.61, *p*<0.001). Patients with a history of falls prior to cirrhosis diagnosis had a 469% increased risk of experiencing a fall compared to those without a fall history (HR: 5.69, 95% CI: 4.93–6.58, *p*<0.001). Patients with HCC had a 45% lower risk of experiencing a fall compared to those without HCC (HR: 0.45, 95% CI: 0.30–0.68, *p*<0.001). Patients with osteoporosis had a 49% increased risk of experiencing a fall (HR: 1.49, 95% CI: 1.21–1.84, *p*<0.001). Regarding high-risk medications, patients who were chronically on diuretics, benzodiazepines, and antidepressants had a 21% (HR: 1.21, 95% CI: 1.06–1.37, *p*=0.004), 57% (HR: 1.57, 95% CI: 1.38–1.77, *p*<0.001), and 14% (HR: 1.15, 95% CI: 1.02–1.29, *p*=0.024) increased risk of falling, respectively (Figure 3).

### Recurrent falls

Of the patients who experienced one fall (n=2997), 38.4% (n=1152) experienced a second during the study period, and the additional fall rate was 1.63 falls pppy (Table 3). Among patients who experienced a second fall, 35% (n=455) experienced the second fall within 1 year of the first. In total, 14.8% (n=171) of the patients with ≥2 falls had a history of falls prior to cirrhosis diagnosis. Women (n=555, 40.2%) were more likely to experience ≥2 falls compared to men (n=597, 37.0%) (*p*<0.001) (Supplemental Table S4, https://links.lww.com/HC9/C454]. Patients who experienced a second fall were more likely to have osteoporosis than those with one fall (6.7% vs. 4.7%, *p*=0.022) (Supplemental Table S5, https://links.lww.com/HC9/C454). Among patients who fell at least twice, patients with no insurance experienced a significantly higher fall rate (6.50 falls pppy) than patients with private insurance (0.644 falls pppy) or Medicare/Medicaid (1.41 falls pppy) (all pairwise comparisons, *p*<0.001). Patients with ALD experienced the highest rate of additional falls (4.04 falls pppy) among the different cirrhosis etiologies (Table 3).

In multivariable regression analysis, patients with Medicare/Medicaid insurance had a 33% increased risk of a second fall compared to patients with private insurance (HR: 1.32, 95% CI: 1.05–1.67, *p*=0.019). Patients with a fall history prior to cirrhosis diagnosis had a 50% increased risk of experiencing a second fall compared to those without a fall history (HR: 1.50, 95% CI: 1.18–1.90, *p*<0.001). Patients with a history of osteoporosis had a 44% increased risk of experiencing a second fall (HR: 1.44, 95% CI: 1.05–1.97, *p*=0.023). Chronic use of benzodiazepines resulted in a 42% increased risk of a second fall (HR: 1.41, 95% CI: 1.15–1.73, *p*<0.001) (Table 5).

**TABLE 5:** Multivariate Cox proportional hazard regression assessing the risk of a second fall

Demographics	HR	95% CI	*p*
Age	1.01	1.01–1.02	<0.001
Sex			
Female	1.00	0.83-1.20	1.000
Race			
Non-Hispanic White	Ref	Ref	Ref
Asian	0.68	0.35–1.35	0.271
Hispanic	0.88	0.70–1.11	0.292
Non-Hispanic Black	0.96	0.77–1.20	0.704
Other	0.43	0.24–0.80	0.008
Insurance			
Private	Ref	Ref	Ref
Medicare/Medicaid	1.32	1.05–1.67	0.019
No insurance	1.40	0.97–2.03	0.076
Other	1.13	0.82–1.57	0.451
Etiology			
MASLD	Ref	Ref	Ref
MetALD	1.02	0.79–1.32	0.861
ALD	1.14	0.76–1.71	0.517
HepB	0.73	0.35–1.52	0.403
HepC	0.91	0.67–1.24	0.551
Two or more	0.83	0.63–1.09	0.185
Other (genetic, cardiac, cholestatic, other)	0.83	0.58–1.20	0.328
Unspecified	<0.001	0.00-∞	0.990
Decompensated at baseline	0.87	0.72–1.05	0.148
Charlson comorbidity index category			
None	Ref	Ref	Ref
Mild	0.60	0.20–1.71	0.334
Moderate	0.57	0.20–1.61	0.286
Severe	0.57	0.21–1.55	0.269
Frailty			
Low frailty	Ref	Ref	Ref
Intermediate frailty	1.31	1.05–1.64	0.019
severe frailty	1.51	1.16–1.96	0.002
HCC status	1.16	0.59–2.27	0.658
Fall history	1.50	1.18–1.90	<0.001
Malnutrition	1.01	0.75–1.36	0.950
Sarcopenia	5.96	0.80–44.56	0.082
Osteoporosis	1.44	1.05–1.97	0.023
diuretics	1.08	0.88–1.33	0.461
Opioids	1.04	0.71–1.53	0.842
Benzodiazepine	1.42	1.15–1.73	<0.001
Antidepressants	1.09	0.89–1.32	0.413

Abbreviations: ALD, alcohol-associated liver disease; MASLD, metabolic dysfunction–associated steatotic liver disease; MetALD, metabolic dysfunction with increased alcohol intake; pppy, per patient per year.

### Frailty

**FIGURE 4:**
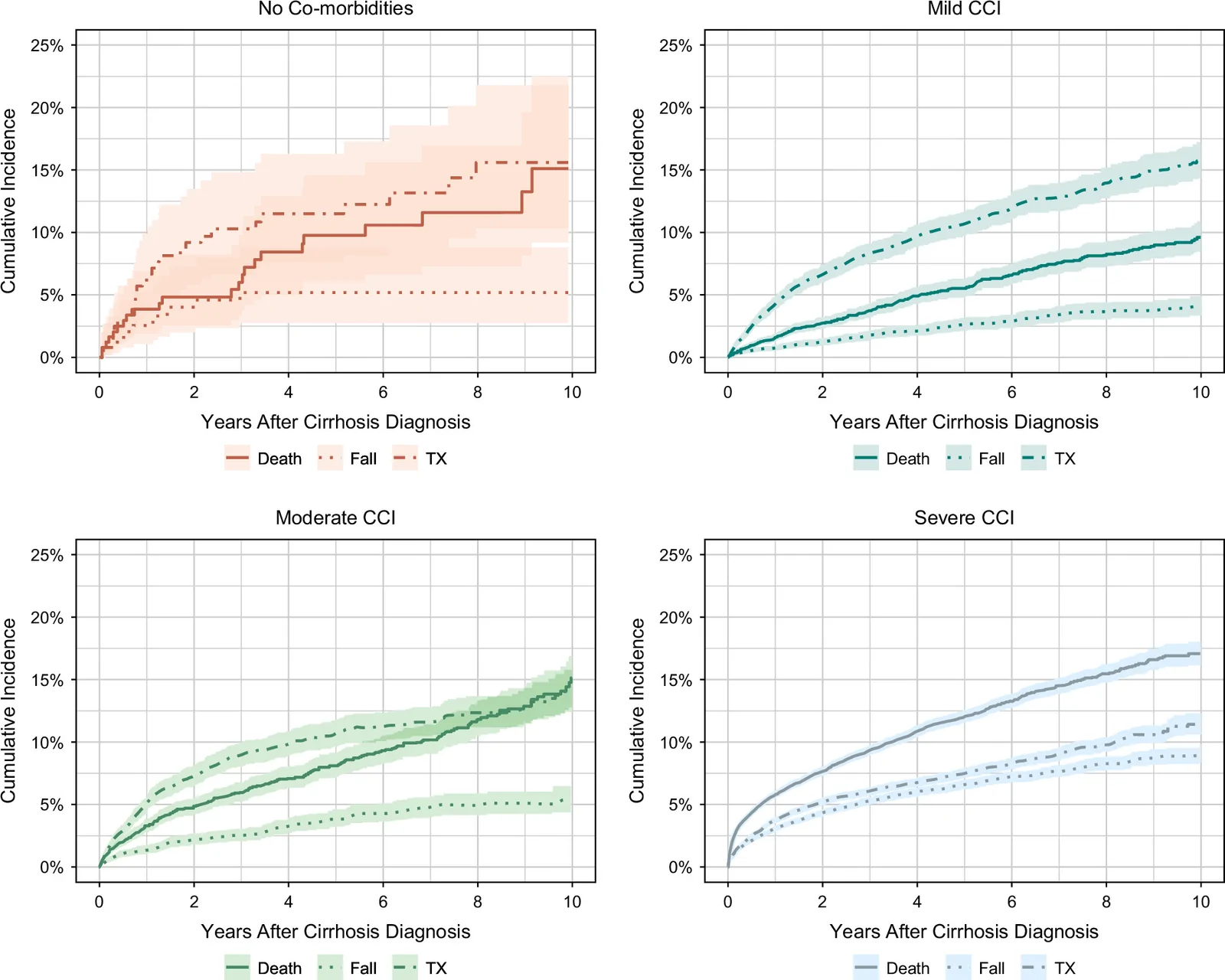
Cumulative incidences of fall, death, and transplant stratified by frailty severity. Abbreviations: CCI, Charlson Comorbidity Index; TX, transplant.

When looking at frailty, a higher proportion of patients with severe frailty experienced a fall compared to those with intermediate or mild frailty (13.37% vs. 9.11% vs. 5.21%, *p*<0.001). Patients with severe frailty experienced higher fall rates than patients with intermediate or mild frailty (1.35 vs. 0.47 vs. 0.09 falls pppy; *p*=0.001). After adjusting for clinicodemographic factors, patients with intermediate and severe frailty had a 71% and 137% increased risk of experiencing a fall compared to patients with mild frailty, respectively (HR: 1.71, 95% CI: 1.49–1.96, *p*<0.001; HR: 2.37, 95% CI: 2.01–2.80, *p*<0.001). Cumulative fall incidence rates increased as frailty increased (Figure 4). Patients with severe frailty had a 1-year cumulative fall incidence of 8.8% (95% CI: 7.9%–9.8%) compared to 3.7% (95% CI: 3.3%–4.2%) for those with intermediate frailty, and 1.1% (95% CI: 1.0%–1.3%) for those with mild frailty. This cumulative fall incidence pattern continues throughout the study period, culminating in a 10-year fall incidence of 19.6% (severe), 11.4% (intermediate), and 5.2% (mild) (Figure 4). When assessing fall recurrence, patients with severe and intermediate frailty had an 51% increased risk (HR: 1.51, 95% CI: 1.16–1.96, *p*=0.002) and a 31% increased risk (HR: 1.31, 95% CI: 1.05–1.64, *p*=0.019) of experiencing a second fall, respectively, compared to patients with mild frailty (Table 5).

## DISCUSSION

In our longitudinal study of patients with cirrhosis across a large metropolitan area, 7% of patients experienced a fall during the study period. The patients at highest risk of experiencing a single or recurrent fall were those with a diagnosis of MetALD or ALD, intermediate or severe frailty, and those with a fall history prior to cirrhosis diagnosis. One in 3 patients who fell a second time experienced their second fall within 1-year, which emphasizes the negative impact a single fall can have on a patient’s trajectory. Consistent with prior literature, fall risk was highest among patients who experienced falls prior to cirrhosis diagnosis.^32^ Severe frailty was the strongest risk factor associated with having a single fall (HR: 2.37). Prior fall history (HR: 1.50) and severe frailty (HR: 1.51) were the strongest risk factors for experiencing a second fall. For patients with severe frailty, the risk of a first fall was 137% higher than for patients with mild frailty, the risk of a second fall was 51% higher than for patients with mild frailty, and the cumulative incidence of a fall over 10 years was 19.6%.

Frailty has increasingly been recognized as a prognostic factor worthy of careful consideration for patients with cirrhosis, given its correlation with hospitalization rates, liver transplant waitlist mortality, and disease progression.^10^,^33^ While assessing the frailty of patients with cirrhosis has emerged as a critical aspect of their care, our study calls attention to an important mechanism through which frailty may develop and progress. Our finding that frailty is associated with increased risk of experiencing a first and a second fall among patients with cirrhosis is consistent with prior research in elderly patients demonstrating that falls often result in debilitating injuries that can increase frailty;^34^ the linkage is so strong that some studies have even used fall risk to estimate frailty.^35^ A study by Tapper et al^12^ shows that frailty is associated with increased fall risk among patients with cirrhosis. Frailty was more predictive of fall risk than disease severity, assessed through baseline decompensation status and MELD-Na score, and comorbidity profile, described using CCI category.

Our finding of patients with Medicaid/Medicare and patients with an alcohol-associated cirrhosis etiology having an increased fall risk reinforces the findings of a retrospective study that used a national dataset of emergency department visits to investigate fall-related injuries among patients with cirrhosis.^36^ In this study, patients with cirrhosis who presented with a fall were more likely to have Medicaid than those without cirrhosis (20.7% vs. 12.5%), and multivariable analysis revealed a significant association between falls resulting in severe injury and alcohol abuse (OR: 1.28).^36^ However, neither study analyzed large datasets that represented the full range of care settings. Other studies of falls in cirrhosis have used small cohorts, involved foreign patient populations, or focused on specific cirrhosis subpopulations.^12^,^23^,^36^,^37^

Each risk factor identified in this study can be used to direct resources toward patients most at risk of falling. Patients with MetALD, ALD, and those with ≥2 cirrhosis etiologies were at increased risk of experiencing a fall compared to other cirrhosis etiologies. These findings highlight that patients with MetALD have a risk profile for falls that is more similar to those with ALD than those with the isolated metabolic syndrome seen in MASLD.^18^ Therefore, patients with MetALD may benefit from additional surveillance and education about appropriate safety measures. Patients who fell were more likely to have high comorbidities, more likely to have psychological/cognitive/substance use disorders, were over twice as likely to carry a diagnosis of osteoporosis and were more likely to use high-risk medications at baseline. These findings can be used to identify patients with a clinical and pharmaceutical phenotype that increases their fall risk and to provide individualized counseling on how to mitigate this risk. Patients with a fall history prior to cirrhosis diagnosis were >3 times more likely to experience a fall than those without a fall history. This trend can help clinicians provide individualized counseling on fall prevention when establishing care with patients with cirrhosis who admit to falling previously, which may include encouraging environmental modifications or placing early referrals. Most falls that patients experienced were in outpatient settings; modifications in the home and community may optimize risk reduction. Given that 81% of falls lead to a documented injury and 42% of falls were associated with a hospitalization, decreasing the fall burden in this population may improve the health of individual patients while also decreasing expensive health care utilization.

A strength of this study is its longitudinal assessment of falls in patients with cirrhosis in a large, retrospective cohort using EMR data across diverse clinical settings (eg, emergency, inpatient, and outpatient care). A fall in this dataset can be contextualized in relation to the date of cirrhosis diagnosis—rather than the date of study enrollment—allowing for time-to-event modeling. However, this study does have some noteworthy limitations. First, due to the limitations of the aggregated retrospective dataset and the imprecise timing of clinical documentation, we were unable to use time-varying covariates in our analytic models. While it is challenging to presume that baseline covariates, such as decompensation status or comorbidities, will remain unchanged during a long follow-up period, this analysis allows for a more conservative estimate of the impact of these variables on the long-term risk of falls. The data presented here suggest a relationship between predictive baseline covariates and falls and may serve as the foundation for future work that provides deeper, prospective insight into the impact of specific covariates on these outcomes.

Only documented falls in the EMR were analyzed, which represent falls that warranted medical evaluation and were captured by ICD or CPT codes in the EMR. Hence, the number of falls included in our analysis may be an underestimation of the true incidence of total falls in this population. This is further affirmed by our finding that 81% of falls were associated with an injury, which indicates that our hospital-based data largely do not capture noninjurious falls. One can presume that falls that do not result in an injury may not prompt patients to seek care. While this may limit the generalizability of our analysis, the most clinically significant falls are those requiring medical attention, and those are the falls captured here.^12^ Given that the data presented here may represent the lower bound of a patient’s fall burden, these findings may be even more useful when counseling patients. In addition, the use of the Hospital Frailty Risk Score (HFRS) to assess frailty has limitations. Although HFRS has been shown to correlate well with objective frailty measures and to be very sensitive for identifying physical frailty, its lack of validation in a cirrhosis-specific cohort, the possibility of redundant ICD codes, and lack of inclusion of key frailty cirrhosis markers, such as sarcopenia,^26^ have led to the predominance of other cirrhosis frailty scores (such as the Liver Frailty Index) and even prompted recent attempts to modify the HFRS.^38^ However, the HFRS remains a frailty score that has been used in a wide variety of cirrhosis populations^10^,^28^,^39^ and has been shown to have better predictive power than other comorbidity measures, such as the Charlson Comorbidity Index and even the Cirrhosis-Specific Co-Morbidity Score.^40^ Using this score here, coupled with the CCI and the addition of sarcopenia, osteoporosis, and malnutrition, adds significant value to our analysis.^25^,^39^ It is challenging to ascertain the exact cause and timing of an injury or a hospitalization as it relates to a fall, given the unreliable and likely imprecise timing of code entry into our dataset. Using a 30-day window around the fall code, though reasonably supported by prior literature,^30^ may overestimate or underestimate the true burden of injuries and hospitalization. Lastly, we do not have information on the proportion of patients with ALD who have active alcohol use disorder or the amount of current alcohol use, which may confound the etiology-specific results reported here.

Characterizing the patients most at risk of falling within the aging cirrhosis population has the potential to inform targeted interventions that improve patient morbidity and health care expenditure. Previous work has shown that interventions such as regular structured exercise,^41^ deprescribing high-risk medications^42^ (eg, anticholinergics, antidepressants, opioids), improving vision^43^ (eg, cataract surgery), and modifying footwear have the potential to decrease a patient’s risk of falling.^44^ Adapting such interventions to patients with cirrhosis may prove to be clinically impactful. Previous studies have focused on falls as an outcome of interest. While falls are an important outcome, the data presented here suggest they may also be key drivers of future falls and, therefore, increased health care utilization. Given that $50 billion USD was spent on care for falls among the elderly in 2015 and $32.5 billion USD was spent on cirrhosis care in 2016,^17^,^45^,^46^ patients with cirrhosis who fall represent a costly and resource-intensive subpopulation worthy of targeted risk mitigation.

In conclusion, our study identified risk factors for falls in patients with cirrhosis with no insurance or Medicare/Medicaid, patients with MetALD or ALD, frail patients, and patients with a history of falling. In the future, this information may be incorporated into algorithms that trigger additional patient education on reducing fall risk or suggest referrals to specialists in an automated fashion. While more work is needed to understand which fall-prevention interventions are effective in the cirrhosis population, this study contributes to our understanding of where and with whom to accelerate these efforts.

## Supplementary Material
