# Supplementary material for: The rate of and risk factors for falls among patients with cirrhosis: A multicenter study
Source: Hepatol Commun. 2026 Aug 20;10(9):e01019. doi: 10.1097/HC9.0000000000001019 (PMC13496322; doi:10.1097/HC9.0000000000001019)
Supplement: Supplementary file 1 [file hc9-10-e01019-s001.docx]

**Supplementary Material**

**Supplementary Table S1**: Cirrhosis Diagnosis Codes

| **Cirrhosis Definitions** | | | |
| --- | --- | --- | --- |
|  |  | ICD-9 | 571.2, 571.5, 571.6 |
|  |  | ICD-10 | K70.30, K70.31, K74.60, K74.69, K74.3, K74.4, K74.5 |
|  | **Clinical Definitions** | | |
|  | HCC | ICD-9 | 155.0 |
|  |  | ICD-10 | C22.0, C22.7, C22.8, C22.9 |
|  |  | CPT | 79445 |
|  | **Decompensation Definitions** |  |  |
|  | Hepatic encephalopathy | ICD-9 | 572.2, 070.0, 070.2xx, 070.4xx, 070.6, 348.3xx |
|  |  | ICD-10 | B15.0, B16.0, B16.2, B17.11, B19.0, B19.11, B19.21, K72.11, K72.90, K72.91, K76.82 |
|  |  | Medications | lactulose, lactitol, rifaximin |
| Variceal Bleeding | | ICD-9 | 456.0, 456.20, 578.xx |
|  | | ICD-10 | I85.11, I85.01, K92.0, K92.1, K92.2 |
|  | | CPT | 43243, 43244, 43255 |
| Ascites | | ICD-9 | 789.59 , 511.89 , 789.5 |
|  | | ICD-10 | K70.11, K70.31, K71.51, R18.8, J90, J94.8, R18.0 |
|  | | Medications | eplerenone, spironolactone, amiloride, furosemide, torsemide, bumetanide |
|  | | CPT | 49082, 49083 |
| SBP | | ICD-9 | 567.xx |
|  | | ICD-10 | K65.2, K65.0, K65.9 |
| HRS | | ICD-9 | 572.4 |
|  | | ICD-10 | K91.83, K76.7 |
| HPS | | ICD-9 | 573.5 |
|  | | ICD-10 | K76.81 |
|  |  |  |  |
|  | **Cirrhosis Etiologies** | | |
|  | Hepatitis B Virus | ICD-9 | 070.2xx, 070.3xx |
|  |  | ICD-10 | V02.61, B16.xx, B18.0, B18.1, B19.9, B19.10, B19.11, |
|  | Hepatitis C Virus | ICD-9 | 070.41, 070.44, 070.51, 070.54, 070.70, 070.71 |
|  |  | ICD-10 | V02.62, B17.10, B17.11, B18.2, B19.20, B19.21 |
|  | Alcohol | ICD-9 | 571.1, 571.2, 571.3, 291.xx, 303.xx |
|  |  | ICD-10 | K70.9, K70.10, K70.11, K70.30, K70.31, F10.x |
|  | Primary Biliary Cholangitis | ICD-9 | 571.6 |
|  |  | ICD-10 | K74.3, K74.4, K74.5 |
|  | Primary Sclerosing Cholangitis | ICD-10 | K83.01, K83.09 |
|  | Autoimmune | ICD-9 | 571.42 |
|  |  | ICD-10 | K75.4 |
|  | Cardiac | ICD-9 | 573.0 |
|  |  | ICD-10 | K76.1 |
|  | Genetic | ICD-9 | 273.4, 275.01, 275.1, 275.03 |
|  |  | ICD-10 | P78.81, E83.110, E83.01, E88.01 |
|  | Other/Unspecified | ICD-9 | 571.8, 571.9, 571.5 |
|  |  | ICD-10 | K74.60, K74.69 |
|  | MASLD/MASH | ICD-9 | 571.8/571.9/571.5 + comorbidities of interest |
|  |  | ICD-10 | K76.0 OR K75.81 OR K74.60/K74.69 + comorbidities of interest |
|  |  | ICD-10 | Any other cirrhosis inclusion code that is not classified as a different etiology + Obesity(BMI>30)/DM/HTN/Dyslipidemia codes |
|  | Comorbidities for MASLD/MASH definition | Comorbidities of interest | Obesity or BMI≥30 |
|  |  |  | Hypertension: "Hypertension uncomplicated", "Hypertension complicated" |
|  |  |  | Diabetes: "Diabetes uncomplicated", "Diabetes complicated" |
|  |  |  | Dyslipidemia |
|  | MetALD |  | MASLD/MASH + ALD ICD codes |
|  | Obesity | ICD-9 | 278.0xx |
|  |  | ICD-10 | E66.xx |
|  | Diabetes, and/or diabetic without complications | ICD-9 | 250.0x, 250.1x, 250.2x, 250.3x, 250.8x, 250.9x, 648.00, 775.1 |
|  |  | ICD-10 | E10.0x, E10.1x, E10.6x, E10.8x, E10.9x, E11.0x, E11.1x, E11.6x, E11.8x, E11.9x, E12.0x, E12.1x, E12.6x, E12.8x, E12.9x, E13.0x, E13.1x, E13.6x, E13.8x, E13.9x, E14.0x, E14.1x, E14.6x, E14.8x, E14.9x |
|  | Diabetes, and/or diabetic with complications | ICD-9 | 250.4x, 250.5x, 250.6x, 250.7x |
|  |  | ICD-10 | E10.2x, E10.3x, E10.4x, E10.5x, E10.7x, E11.2x, E11.3x, E11.39, E11.4x, E11.5x, E11.7, E12.2, E12.3, E12.4, E12.5, E12.7, E13.2x, E13.3x, E13.4x, E13.5x, E13.7, E14.2, E14.3, E14.4, E14.5, E14.7 |
|  | Hypertension without complication | ICD-9 | 401.x, 642.0x |
|  |  | ICD-10 | I10 |
|  | Hypertension with complication | ICD-9 | 402.x, 403.x, 404.x, 405.x, 642.1x, 642.2x, 642.7x, 642.9x, |
|  |  | ICD-10 | I11.x, I12.x, I13.x, I15.x |
|  | Dyslipidemia | ICD-9 | 272.x, 277.7 |
|  |  | ICD-10 | E78.x, E88.81 |

Supplementary Table S2: Co-Morbidity Diagnosis Codes and Medications

| **Co-Morbidity Definitions** | | | |
| --- | --- | --- | --- |
|  | Coronary artery disease | ICD-9 | 410-414 |
|  |  | ICD-10 | I20-I25 |
|  | Cerebrovascular disease | ICD-9 | 362.34, 430-438 |
|  |  | ICD-10 | G45, G46, H34.0, I60-I69 |
|  | Congestive heart failure | ICD-9 | 402.01, 402.11, 402.91, 404.01, 404.03, 404.11, 404.13, 404.91, 404.93, 428 |
|  |  | ICD-10 | I11.0, I13.0, I13.2, I25.5, I42.0, I42.5, I42.6, I42.7, I42.8, I42.9 |
|  | Moderate or severe renal disease | ICD-9 | 403.01, 403.11, 403.91, 404.02, 404.03, 404.12, 404.13, 404.92, 404.93, 582, 583.0-583.7, 585, 586, 588.0, V42.0, V45.1, V56. |
|  |  | ICD-10 | I12.0, I13.1, N03.2-N03.7, N05.2-N05.7, N18, N19, N25.0, Z49.0-Z49.2, Z94.0, Z99.2 |
| Diabetes mellitus | | ICD-9 | 250.0-250.9 |
|  | | ICD-10 | E10-E14 |
| Non-HCC Malignancy | | ICD-9 | 140-154, 156-199, 200-209, 230-239 |
|  | | ICD-10 | C00-C21, C23-C96, D00-09, D37-49 |
| Psychological disorders/Cognitive disorders/substance use disorders | | ICD-9 | 291-319 |
|  | | ICD-10 | F04-F99 |
| Dementia | | ICD-9 | 290, 294.1, 294.2, 331 |
|  | | ICD-10 | F01-F03 |
| Osteoporosis | | ICD-9 | 733.0 |
|  | | ICD-10 | M80, M81 |
| Malnutrition | | ICD-9 | 260-269 |
|  | |  | E40-E46 |
| Sarcopenia | | ICD-9 | --- |
|  | |  | M62.84 |
|  |  |  |  |
|  | **Medication Class** | | |
|  | Diuretics | Medications | Eplerenone, spironolactone, amiloride, furosemide, torsemide, bumetanide, |
|  | Opioids | Medications | Morphine, oxycodone, hydromorphone, buprenorphine, codeine, fentanyl, hydrocodone, levorphanol, meperidine, methadone, tramadol |
|  | Benzodiazepines | Medications | Lorazepam, diazepam, clonazepam, alprazolam, zolpidem, chlordiazepoxide, |
|  | Antidepressant | Medications | Amitriptyline, amoxapine, clomipramine, desipramine, doxepin, imipramine, nortriptyline, protriptyline, trimipramine, desvenlafaxine, duloxetine, citalopram, escitalopram, fluoxetine, milnacipran, venlafaxine, fluvoxamine, paroxetine, sertraline, |

**Supplementary Table S3**: Fall and Injury Diagnosis Codes

|  | ICD-9-CM |  | ICD-10-CM |  |  |
| --- | --- | --- | --- | --- | --- |
|  | Code | Identifier | Code | Identifier |  |
| External Injury Codes | E880.0-E886.9,  E888 | Unintentional Fall | V00.11-V00.89  W00-W17  W18.1, W18.2, W18.3  W19 | Unintentional Falls  Unspecified Fall | |
| Injury Diagnosis Codes | 800-904, 910-957 | Injury to body part | S00-S99  T07-T34  T84.04 and M97 | Injury to body part  Foreign bodies, burns, corrosions, frostbite  Prosthetic joint injuries |  |
|  | 960-989,  995.5, 995.80-995.85  990-994 | Poisoning and toxic effects  Certain adverse effects, not classified elsewhere  Other unspecified effects of external causes | T36-T76 | Drug poisoning, toxic substance effects, and other unspecified effects |  |
|  | 958-959  905-909.2, 909.4, 909.9 | Certain complications of trauma  Late effects of injury | T79 | Complications of trauma |  |

Obtained from:

Moreland BL, Burns ER, Haddad YK. National rates of non-fatal emergency department visits and hospitalisations due to fall-related injuries in older adults 2010-2014 and 2016: transitioning from ICD-9-CM to ICD-10-CM. Inj Prev. 2021 Mar;27(S1):i75-i78. doi: 10.1136/injuryprev-2019-043516. PMID: 33674338; PMCID: PMC7948188.

Supplementary Table S4: Patient demographics comparing those who fall ≥ 2 times to those who experienced only 1 fall

|  | Demographics | Total Cohort with ≥ 1 Fall | Patients with ≥2 Falls | % (by row) | Patients with Only 1 Fall | % (by row) | P-value |
| --- | --- | --- | --- | --- | --- | --- | --- |
|  | TOTAL (n) | 2997 | 1152 | 38.4% | 1845 | 61.6% |  |
|  | Age (mean)  {median} [IQR] | (60.7){60.9}  [52.9-69.1] | (61.1){60.8}  [53.2-70] |  | (60.5){61}  [52.6-68.6] |  | 0.271 |
|  | Follow-up Time (mean) {median} [IQR] | (2.2){1.5}  [0.37-3.47] | (2.8){2.1}  [0.89-4.25] |  | (1.9){1.13}  [0.20-2.97] |  | <0.001 |
| SEX (n) | |  |  |  |  |  | <0.001 |
| Men | | 1615 | 597 | 37.0% | 1018 | 63.0% |  |
| Women | | 1382 | 555 | 40.2% | 827 | 59.8% |  |
|  | Race |  |  |  |  |  | <0.001 |
|  | Asian | 64 | 19 | 29.7% | 45 | 70.3% |  |
|  | Hispanic | 712 | 289 | 40.6% | 423 | 59.4% |  |
|  | Non-Hispanic Black | 677 | 284 | 42.0% | 393 | 58.1% |  |
|  | Non-Hispanic White | 1425 | 534 | 37.5% | 891 | 62.5% |  |
|  | Other | 119 | 26 | 21.9% | 93 | 78.2% |  |
|  | Insurance  status |  |  |  |  |  | 0.035 |
|  | Private | 642 | 213 | 33.1% | 429 | 66.9% |  |
|  | Medicare/  Medicaid | 1668 | 650 | 39.6% | 1018 | 60.4% |  |
|  | no insurance | 247 | 112 | 45.3% | 135 | 54.7% |  |
|  | Other | 440 | 177 | 40.2% | 263 | 59.8% |  |
| Etiology | |  |  |  |  |  | 0.084 |
| MASLD | | 618 | 235 | 38.0% | 383 | 62.0% |  |
| MetALD | | 864 | 351 | 40.6% | 513 | 59.4% |  |
| ALD | | 170 | 52 | 30.6% | 118 | 69.4% |  |
| HCV | | 321 | 122 | 38.0% | 199 | 62.0% |  |
| HBV | | 54 | 16 | 29.6% | 38 | 70.4% |  |
| Two or more | | 746 | 292 | 39.1% | 454 | 60.9% |  |
| Unspecified | | 7 | 0 | 0.00% | 7 | 100.0% |  |
| Other | | 217 | 84 | 38.7% | 133 | 61.3% |  |
|  | Compensation Status |  |  |  |  |  | 0.9 |
|  | Compensated at Baseline | 1678 | 644 | 38.3% | 837 | 60.56% |  |
|  | Decompensated at Baseline | 1319 | 508 | 38.5% | 1008 | 62.4% |  |
|  | HE Status |  |  |  |  |  | 0.9 |
|  | HE at Baseline | 487 | 153 | 31.4% | 334 | 68.58% |  |
|  | No HE at Baseline | 2510 | 999 | 39.8% | 1511 | 60.2% |  |
| Frailty | |  |  |  |  |  | 0.519 |
| Mild | | 1406 | 528 | 37.6% | 878 | 62.5% |  |
| Intermediate | | 906 | 349 | 38.5% | 557 | 61.5% |  |
| Severe | | 685 | 275 | 40.2% | 410 | 59.9% |  |
|  | MELD-Na (Mean score) {IQR} [% with calculable score] | (16) {10-24} [63.8%] | (17.0) {10-23} [61.6%] |  | (15.0) {10-24} [65.2%] |  |  |
| Fall History | | 400 | 171 | 42.8% | 229 | 57.3% | 0.065 |
|  | HCC | 69 | 19 | 27.5% | 50 | 72.5% | 0.046 |
|  |  |  |  |  |  |  |  |

* HE = hepatic encephalopathy

**Supplementary Table S5**: Patient co-morbidities comparing those who fall ≥ 2 times to those who experienced only 1 fall

|  | Demographics | Total Cohort with ≥1 fall (N=2,997) | Patients with ≥2 Falls(N=1,152) | Patients with only 1 Fall (N=1,845) | P-value |
| --- | --- | --- | --- | --- | --- |
| Charlson Comorbidity Index (CCI) | |  |  |  |  |
| Continuous, median (IQR) | | 9 (6-13) | 9 (6-13) | 9 (6-13) | 0.6 |
| 0: No comorbidities, N (COL %) | | 20 (0.67%) | 7 (0.61%) | 13 (0.70%) | 0.8 |
| 1-2: Mild, N (col %) comorbidity | | 284 (9.5%) | 109 (9.5%) | 175 (9.5%) |  |
| 3-4: Moderate comorbidity (col %) | | 290 (9.7%) | 105 (9.1%) | 185 (10.0%) |  |
| ≥5: Severe Comorbidity (col %) | | 2403 (80.2%) | 931 (80.8%) | 1472 (79.8%) |  |
| Specific comorbidities | |  |  |  |  |
| Coronary Artery Disease, n (col %) | | 593 (19.8%) | 239 (20.7%) | 354 (19.2%) | 0.3 |
| Cerebrovascular disease, N (col %) | | 210 (7.0%) | 84 (7.3%) | 126 (6.8%) | 0.7 |
| Congestive heart failure, N (col %) | | 492 (16.4%) | 209 (18.1%) | 283 (15.3%) | 0.049 |
| Moderate or severe kidney disease, N (col %) | | 240 (8.0%) | 90 (7.8%) | 150 (8.1%) | 0.8 |
| Diabetes Mellitus, N (col %) | | 1015 (33.9%) | 413 (35.9%) | 602 (32.6%) | 0.076 |
| Non-HCC Cancer, N (col %) | | 410 (13.7%) | 164 (14.2%) | 246 (13.3%) | 0.5 |
| Psychological disorder/cognitive disorder/substance use disorder, n (col %) | | 1556 (51.9%) | 619 (53.7%) | 937 (50.8%) | 0.13 |
| Dementia, N (col %) | | 111 (3.7%) | 36 (3.1%) | 75 (4.1%) | 0.2 |
| Osteoporosis, N (col %) | | 163 (5.4%) | 77 (6.7%) | 86 (4.7%) | 0.022 |
| Malnutrition, N (col %) | | 278 (9.3%) | 102 (8.9%) | 176 (9.5%) | 0.6 |
| Sarcopenia, N (col %) | | 5 (0.17%) | 2 (0.17%) | 3 (0.16) | 0.9 |
| medications | |  |  |  |  |
| Baseline Diuretic use, N (col %) | | 776 (25.9%) | 320 (27.8%) | 456 (24.7%) | 0.069 |
| Baseline opioid use, N (col %) | | 116 (3.9%) | 52 (4.5%) | 64 (3.5%) | 0.20 |
| Baseline benzodiazepine use, N (col %) | | 828 (27.6%) | 316 (27.4%) | 512 (27.8%) | 0.021 |
| Baseline antidepressant use, N (col %) | | 995 (33.2%) | 394 (34.2%) | 601 (32.6%) | 0.40 |

**Supplementary Table S6**: Sensitivity analysis comparing Fine Gray model to cause-specific hazard model (CSHM) assessing risk factors for experiencing a first fall

|  | Variable | Fine Gray Hazard ratio | 95% CI | P-value | CSHM HAzard Ratio | 95% CI | P-value |
| --- | --- | --- | --- | --- | --- | --- | --- |
| Age | | 1.02 | 1.02,1.03 | <0.001 | 1.02 | 1.02,1.03 | <0.001 |
| Sex | |  |  |  |  |  |  |
| Female | | 1.17 | 1.05-1.30 | 0.004 | 1.15 | 1.03,1.28 | 0.011 |
| Race | |  |  |  |  |  |  |
| Non-Hispanic white | | REF | REF | REF | REF | REF | REF |
| Asian | | 0.67 | 0.45,0.99 | 0.046 | 0.70 | 0.47,1.03 | 0.071 |
| Hispanic | | 1.20 | 1.05,1.30 | 0.007 | 1.17 | 1.03,1.34 | 0.020 |
| Non-Hispanic Black | | 0.87 | 0.75,1.00 | 0.042 | 0.81 | 0.71,0.93 | 0.003 |
| Other | | 0.46 | 0.35,0.61 | <0.001 | 0.47 | 0.35,0.61 | <0.001 |
| Insurance | |  |  |  |  |  |  |
| Private | | REF | REF | REF | REF | REF | REF |
| Medicare/  Medicaid | | 1.42 | 1.24,1.64 | <0.001 | 1.40 | 1.23,1.61 | <0.001 |
| No insurance | | 1.61 | 1.29,2.00 | <0.001 | 1.57 | 1.26,1.96 | <0.001 |
| Other | | 0.96 | 0.80,1.15 | 0.636 | 0.96 | 0.80,1.16 | 0.690 |
| Etiology | |  |  |  |  |  |  |
| MASLD | | REF | REF | REF | REF | REF | REF |
| MetALD | | 1.50 | 1.29,1.74 | <0.001 | 1.47 | 1.26,1.71 | <0.001 |
| ALD | | 1.63 | 1.32,2.00 | <0.001 | 1.68 | 1.36,2.07 | <0.001 |
| HepB | | 1.00 | 0.67,1.50 | 0.988 | 0.99 | 0.65,1.50 | 0.953 |
| HepC | | 1.03 | 0.85,1.23 | 0.784 | 0.99 | 0.82,1.19 | 0.897 |
| Two or more | | 1.24 | 1.05,1.45 | 0.011 | 1.22 | 1.04,1.44 | 0.017 |
| Other (Genetic, Card, Chol, Other) | | 0.91 | 0.73,1.13 | 0.401 | 0.90 | 0.73,1.12 | 0.360 |
| Unspecified | | 0.32 | 0.14,0.72 | 0.006 | 0.33 | 0.14,0.73 | 0.007 |
| Decompensation status at Baseline | |  |  |  |  |  |  |
| Decompensated | | 0.82 | 0.74,0.93 | 0.001 | 0.97 | 0.86,1.08 | 0.553 |
| Charlson co-morbidity index category | |  |  |  |  |  |  |
| None | | REF | REF | REF | REF | REF | REF |
| mild | | 0.49 | 0.27,0.92 | 0.026 | 0.53 | 0.29,0.99 | 0.047 |
| moderate | | 0.64 | 0.34,1.18 | 0.155 | 0.68 | 0.37,1.27 | 0.225 |
| severe | | 0.68 | 0.37,1.25 | 0.212 | 0.73 | 0.40,1.34 | 0.310 |
| Frailty | |  |  |  |  |  |  |
| Low Frailty | | REF | REF | REF | REF | REF | REF |
| Intermediate Frailty | | 1.69 | 1.47,1.95 | <0.001 | 1.71 | 1.49,1.96 | <0.001 |
| severe Frailty | | 2.17 | 1.83,2.58 | <0.001 | 2.37 | 2.01,2.80 | <0.001 |
| HCC Status | | 0.36 | 0.24,0.54 | <0.001 | 0.45 | 0.30,0.68 | <0.001 |
| Fall History | | 5.65 | 4.85,6.58 | <0.001 | 5.69 | 4.93,6.58 | <0.001 |
| Malnutrition | | 1.15 | 0.95,1.38 | 0.144 | 1.16 | 0.97,1.39 | 0.100 |
| Sarcopenia | | 0.63 | 0.09,4.51 | 0.647 | 1.03 | 0.14,7.36 | 0.974 |
| Osteoporosis | | 1.43 | 1.15,1.77 | 0.001 | 1.49 | 1.21,1.84 | <0.001 |
| diuretics | | 1.17 | 1.03,1.33 | 0.019 | 1.21 | 1.06,1.37 | 0.004 |
| Opioids | | 0.98 | 0.75,1.27 | 0.854 | 0.98 | 0.75,1.26 | 0.850 |
| Benzodiazepine | | 1.53 | 1.35,1.75 | <0.001 | 1.57 | 1.38,1.77 | <0.001 |
| Antidepressants | | 1.06 | 0.95,1.20 | 0.301 | 1.14 | 1.02,1.29 | 0.024 |

**Supplementary Table S7**: Sensitivity analysis comparing Fine Gray model to cause-specific hazard model (CSHM) assessing risk factors for experiencing a second fall

|  | Variable | Fine Gray Hazard ratio | 95% CI | P-value | CSHM HAzard Ratio | 95% CI | P-value |
| --- | --- | --- | --- | --- | --- | --- | --- |
| Age | | 1.02 | 1.02-1.03 | <0.001 | 1.01 | 1.01-1.02 | <0.001 |
| Sex | |  |  |  |  |  |  |
| Female | | 1.01 | 0.84-1.21 | 0.900 | 1.00 | 0.83-1.20 | 1.000 |
| Race | |  |  |  |  |  |  |
| Non-Hispanic white | | REF | REF | REF | REF | REF | REF |
| Asian | | 0.64 | 0.33-1.26 | 0.196 | 0.68 | 0.35-1.35 | 0.271 |
| Hispanic | | 0.92 | 0.73-1.15 | 0.452 | 0.88 | 0.70-1.11 | 0.292 |
| Non-Hispanic Black | | 1.00 | 0.80-1.25 | 0.991 | 0.96 | 0.77-1.20 | 0.704 |
| Other | | 0.43 | 0.24-0.77 | 0.005 | 0.43 | 0.24-0.80 | 0.008 |
| Insurance | |  |  |  |  |  |  |
| Private | | REF | REF | REF | REF | REF | REF |
| Medicare/  Medicaid | | 1.33 | 1.06-1.67 | 0.013 | 1.32 | 1.05-1.67 | 0.019 |
| No insurance | | 1.41 | 1.00,2.00 | 0.050 | 1.40 | 0.97-2.03 | 0.076 |
| Other | | 1.14 | 0.83,1.57 | 0.422 | 1.13 | 0.82-1.57 | 0.451 |
| Etiology | |  |  |  |  |  |  |
| MASLD | | REF | REF | REF | REF | REF | REF |
| MetALD | | 1.00 | 0.78-1.29 | 1.000 | 1.02 | 0.79-1.32 | 0.861 |
| ALD | | 1.06 | 0.79-1.59 | 0.793 | 1.14 | 0.76-1.71 | 0.517 |
| HepB | | 0.75 | 0.36-1.57 | 0.447 | 0.73 | 0.35-1.52 | 0.403 |
| HepC | | 0.86 | 0.65-1.15 | 0.317 | 0.91 | 0.67-1.24 | 0.551 |
| Two or more | | 0.77 | 0.59-1.01 | 0.063 | 0.83 | 0.63-1.09 | 0.185 |
| Other (Genetic, Card, Chol, Other) | | 0.79 | 0.56-1.11 | 0.171 | 0.83 | 0.58-1.20 | 0.328 |
| Unspecified | | <0.001 | 0.00-0.00 | <0.001 | <0.001 | 0.00-∞ | 0.990 |
| Decompensation status at Baseline | |  |  |  |  |  |  |
| Decompensated | | 0.80 | 0.66-0.97 | 0.022 | 0.87 | 0.72-1.05 | 0.148 |
| Charlson co-morbidity index category | |  |  |  |  |  |  |
| None | | REF | REF | REF | REF | REF | REF |
| mild | | 0.56 | 0.18,1.72 | 0.309 | 0.60 | 0.20-1.71 | 0.334 |
| moderate | | 0.57 | 0.19,1.73 | 0.317 | 0.57 | 0.20-1.61 | 0.286 |
| severe | | 0.58 | 0.19,1.65 | 0.291 | 0.57 | 0.21-1.55 | 0.269 |
| Frailty | |  |  |  |  |  |  |
| Low Frailty | | REF | REF | REF | REF | REF | REF |
| Intermediate Frailty | | 1.34 | 1.07-1.67 | 0.011 | 1.31 | 1.05-1.64 | 0.019 |
| severe Frailty | | 1.47 | 1.13-1.91 | 0.004 | 1.51 | 1.16-1.96 | 0.002 |
| HCC Status | | 0.78 | 0.41-1.48 | 0.454 | 1.16 | 0.59-2.27 | 0.658 |
| Fall History | | 1.46 | 1.13-1.88 | 0.004 | 1.50 | 1.18-1.90 | <0.001 |
| Malnutrition | | 0.96 | 0.70-1.31 | 0.805 | 1.01 | 0.75-1.36 | 0.950 |
| Sarcopenia | | 3.14 | 0.42-2.36 | 0.265 | 5.96 | 0.80-44.56 | 0.082 |
| Osteoporosis | | 1.50 | 1.10-2.04 | 0.010 | 1.44 | 1.05-1.97 | 0.023 |
| diuretics | | 1.06 | 0.86-1.30 | 0.585 | 1.08 | 0.88-1.33 | 0.461 |
| Opioids | | 1.10 | 0.74-1.64 | 0.649 | 1.04 | 0.71-1.53 | 0.842 |
| Benzodiazepine | | 1.50 | 1.22-1.85 | <0.001 | 1.42 | 1.15-1.73 | <0.001 |
| Antidepressants | | 1.06 | 0.87-1.28 | 0.562 | 1.09 | 0.89-1.32 | 0.413 |

**Figure S1**: Cumulative incidences of fall, death, and transplant stratified by cirrhosis etiology

 The cumulative incidence of falls is displayed for patients with different cirrhosis etiologies: MASLD = metabolic dysfunction-associated steatotic liver disease. MetALD = metabolic dysfunction with increased alcohol intake. ALD = alcohol-associated liver disease. HCV = hepatitis C virus. HBV = hepatitis B virus. Two+ = two or more cirrhosis etiologies. Incidence accounts for the competing risk of death and transplantation (TX), also displayed.

**Figure S2**: Cumulative incidences of fall, death, and transplant stratified by MELD-Na

The cumulative incidence of falls is displayed for patients stratified by MELD-Na score. Incidence accounts for the competing risk of death and transplantation (TX), also displayed.

**Figure S3**: Cumulative incidences of fall, death, and transplant stratified by age category

The cumulative incidence of falls is displayed for patients stratified by age category: <55, 55-65, 65-75, ≥75. Incidence accounts for the competing risk of death and transplantation (TX), also displayed.
